# Detection of Schmallenberg virus antibody in equine population of Northern and Northeast of Iran

**DOI:** 10.14202/vetworld.2018.30-33

**Published:** 2017-01-18

**Authors:** M. Rasekh, A. Sarani, S. H. Hashemi

**Affiliations:** Department of Clinical Sciences, Faculty of Veterinary Medicine, University of Zabol, Zabol, Iran

**Keywords:** horse, Iran, new emerging disease, Schmallenberg virus

## Abstract

**Aim:**

Schmallenberg virus (SBV) is a newly emerging virus in Simbu group that 1^st^ time is reported in 2011 in Germany and now spread to Europe. The clinical signs of infection to this virus are fever, loss of appetite, reduced milk yield and in some cases, diarrhea and in pregnant animals congenital malformations in calves, lambs, and kid goats.

**Materials and Methods:**

In this study for a serologic survey of SBV, blood samples from 200 horse in different rural areas of the northern and northeast of Iran with the high equine population collected and were analyzed using an indirect ELISA test.

**Results:**

Based on our results 5% (n=10) of total 200 samples were positive for SBV antibody and 2% (n=4) was doubtful and 93% (n=186) was negative. There were no significant differences between age and sex and breed properties (p>0.05).

**Conclusion:**

This study demonstrated the presence of antibodies against the SBV on horse populations in Iran. The high population and activity of Culicoides biting midges and their proper living conditions, especially the areas of temperate and humid environmental conditions, are the possible causes of arboviruses related diseases seen in this country.

## Introduction

Schmallenberg virus (SBV) is a newly emerging RNA virus belonging to Simbu serogroup of Orthobunyavirus genus from the Bunyaviridae family and like as other diseases that caused by Simbu serogroup viruses can induce congenital malformations, stillbirth, severe arthrogryposis, torticollis, brachygnathia, hydrocephaly, ataxia, paralysis, muscle atrophy, joint malformations, scoliosis, behavioral abnormalities, and blindness in fetuses and neonates, especially in ruminants. These malformations have been designated arthrogryposis-hydranencephaly syndrome [1-4].

To date primary, SBV reported in domestic ruminants and antibodies in many species of nonruminant’s wild animals including zebras and the babirusa (belonging to the Suidae family). Other reports indicated that Camelidae such as alpacas was positive for SBV antibodies [3-6]. SBV antibodies were also detected in one dog in Sweden [7]. Similar to other Simbu serogroup viruses, researchers have shown that SBV is suspected to be transmitted through Culicoides biting midges [8-10].

In Iran, there is no scientific investigation on SBV, and the aim of this study was to detect antibodies against SBV in horses of Iran for the 1^st^ time.

## Materials and Methods

### Ethical approval

As the study was conducted with the clinical cases, so Ethical Committee approval was not required. The experiments comply with the guidelines laid down by the Institutional Ethical Committee and in accordance with the country law and regulations.

### Study area and period

The study was carried out from July 2014 to September 2015 in different farms of the rural area of the northern and northeast of Iran which has high equine population. The climate properties of the mentioned area characterized as temperate and semi-desert.

### Study method

Blood samples were taken from the jugular vein of 200 healthy horses and were centrifuged in plain tubes at 10,000 rpm for 10 min. Serum was separated and kept in the freezer at −20°C for serological studies. Serum samples were analyzed for detection of SBV antibody using ID vet^®^ SBV indirect multispecies ELISA test kit (ID Screen SBV multispecies ELISA kit; ID vet, Montpellier, France). ELISA reader was used for determining optic density values. According to the manufacturer’s instructions, the S/P percentage was calculated for each sample. Risk factors such as sex, age, breed and the area were taken into consideration.

### Statistical analysis

Statistical analysis was conducted using SPSS 18.0 software. To evaluate the associations between suggested risk factors and SBV seropositivity, Chi-square test and Fisher’s exact test were used. Considering the comparisons of age variable (ordinal) for the result, linear by linear Chi-square test had been applied.

## Results

A sample was considered positive if the calculated percentage was >60% (ID vet) and ≤50% are considered negative and >50% and inferior or equal to 60% are considered doubtful. On this basis from a total of 200 serum samples that analyzed for SBV presence, 5% (n=10) of total samples was positive for SBV antibody, and 2% (n=4) was doubtful, and 93% (n=186) was negative (Table-1). Due to lack of SBV infection reporting in Iran and expression of the results cautiously, the doubtful results of data analysis were considered as negative.

**Table-1:** Risk factors for SBV in 200 horses.

Risk factors	N (%)		Total		p value
	
	Positive (n=10)	Negative	Positive (%)	Negative (%)	
Sex^†^					
Female	6 (8.70)	63 (91.30)	3	31.5	0.097
Male	4 (3.05)	127 (96.95)	2	63.5	
Breed^†^					
Thoroughbred	2 (16.66)	10 (83.34)	1	5	0.286
Turkmen	6 (4.51)	127 (95.49)	3	63.5	
Mix	2 (3.63)	53 (96.37)	1	26.5	
Age^†^					
<2 years	0 (0)	15 (100)	0	7.5	0.253
25 years	8 (4.96)	153 (95.04)	4	76.5	
>5 years	2 (8.33)	22 (91.66)	1	11	
Area^†^					
North Khorasan	7 (6.08)	108 (93.92)	3.5	54	0.522
Razavi Khorasan	3 (3.52)	82 (96.48)	1.5	41	

* p values were >0.05. SBV=Schmallenberg virus

In this study, risk factors such as age, sex, breed, and geographic location were examined, and no statistically significant difference was observed (p>0.05).

## Discussion

SBV infection is a newly emerging infectious disease that may cause huge economic losses to livestock ruminants. Infection with SBV is studied in different species and is detected in cattle, sheep, goat, alpaca, red deer, and water Anatolian buffaloes [5-8]. In cattle, the acute onset of the disease is a typical form and is characterized by obvious clinical signs of fever, diarrhea, and milk yield reduction and that subside with the animal recovering in 2-3 weeks. In sheep and goats, SBV infection is almost without symptoms that transmission of SBV to fetuses in pregnant animals is through placenta causing abortions, stillbirths and a variety of congenital malformations mainly involving the skeletal and nervous systems [11]. Furthermore, the antibodies against SBV were also detected in many species of nonruminant’s animals including dog and zebra [7,12].

In the current study, the ELISA test was used using ID vet^®^ SBV indirect multispecies ELISA test kit for the detection of SBV antibody and 5% (n=10) of total samples was positive for SBV antibody. Based on manufacturer instruction, the kit wells are coated with the recombinant nucleocapsid protein of SBV, thus cross-reaction with other related Simbu serogroups such as *Shuni* (SHUV), *Aino* (AINV), and *Akabane* (AKAV) virus might be interpreted. Based on recent studies indicated that antibodies to AKAV and AINV will not provide protective immunity to SBV [13], the cross-reaction of suspected viruses and resultant provoked antibodies is less probable. Furthermore, the related viruses have not been reported in the equine population of Iran until present date. In the other hand, in a study undertaken to determine the relation between the role of SHUV and clinical signs in horses, the researchers found that the role of the virus in the development of neurological signs is significant [14], while in the current study, in contrary to ruminants, the seropositive horses have not presented clinical signs of orthobunyaviruses related diseases such as abortion or fetal congenital abnormalities in their history. Although there have been several reports on the presence of antibodies against arboviruses in horses, AKAV and AINV are not known to cause illness in these animals [15,16].

The Schmallenberg disease has been simply detected in domestic ruminants such as cattle, sheep, and goats emerged from Europe and extended to turkey in the middle-east but the investigation of SBV antibody among these species has not been made until the present date in Iran, although unpublished data raised from another study which has been performed for the 1^st^ time by the authors of this article, reveals that considerable level of antibody against SBV exists among the ruminant populations of Iran.

The SBV is one of the arboviruses is known as the arthropod-transmitted diseases. The most important of these arthropods are certain species of Culicoides biting midge. Multiple potential factors such as climate change, anthropogenic, and social factors and/or the movement of vectors or their hosts which are infected by the virus will favor the emergence of these diseases [1,2,17,18]. The disease is first described in the border region of Germany and the Netherlands, between August and October 2011 and extended to neighbored countries [11]. On the other hand, another study which has been performed in turkey between 2006 and 2013 indicated that the virus was presented before its detection in 2011. As Culicoides midges can spread infection across national borders [8,10,19] and they are also distributed with high activity, it is expected that the diseases associated with arboviruses have already existed in the neighbor’s countries such as Iran. Presence of diseases associated with arboviruses such as African horse sickness and Bluetongue proves the high population and activity of the vector (Culicoides) in Iran.

The study was performed on the horses’ population of Iran mainly from northern and northeast of the country, and the antibody of SBV was detected in 5% of the horse population. The positive samples were also from northern and northeast of Iran which nearly has the same climate properties, moist, and temperate. Although the true role of climate changes in causing the recent global expansion of the range and distribution of arboviruses remains conjectural [20].

## Conclusion

The published reports revealed no serological evidence of SBV antibody in horses, thus this is the first serological report which detected the SBV antibody in equine population. Despite the reasons mentioned above and detection of antibodies against the SBV, the authors of this article believe that the claim that the disease exists in Iran is very coarse and imprudent, however, a 5% serologic outbreak can significantly increase the risk of new emerging diseases in various animal populations. As the antibodies against SBV is detected for the 1^st^ time in horses in Iran, more solid shreds of evidence are needed to conclude the association between seropositivity and existence of the disease. The necessity of a repetition of ELISA test to express the definite existence of the disease is inevitable and performing the molecular and more accurate serological tests such as PCR and Virus Neutralization test, respectively, should not be underestimated. Due to high population and activity of Culicoides biting midges and their proper living conditions, especially the areas of temperate and humid environmental conditions and proximity to SBV infected countries (Turkey), it is highly expected that the SBV might be infected the livestock ruminants of Iran.

## Authors’ Contributions

MR and AS were in charge of designing the study and writing the manuscript. The samples were taken by AS and SHH. The supervision of the laboratory work performed by MR and AS. All authors have read and approved the final manuscript.
